# Reviewer acknowledgement 2012

**DOI:** 10.1186/1471-2261-13-14

**Published:** 2013-03-19

**Authors:** Tim Shipley

**Affiliations:** 1 BioMed Central, 236 Gray's Inn Road, London, WC1X 8HB, United Kingdom

## Abstract

**Contributing reviewers:**

The editors of BMC Cardiovascular Disorders would like to thank all our reviewers who have contributed to the journal in Volume 12 (2012).

## 

Mehrshad Abbasi

Iran

Rufus Adedoyin

Nigeria

Mehmet Agirbasli

Turkey

Muharrem Akin

Germany

Patience Akinwusi

Nigeria

Ahmet Akyel

Turkey

Mushfique Alam

United Kingdom

Larry Allen

United States of America

Norrina Allen

United States of America

Giuseppe Alloatti

Italy

Wassim Almawi

Bahrain

Sekip Altunkan

Turkey

Gianfranco Amodio

Italy

Peter Anderson

United States of America

Bruno Andrea

Brazil

Fabio Angeli

Italy

Emiliano Angeloni

Italy

Uzair Ansari

Germany

Riitta Antikainen

Finland

Charalambos Antoniades

United Kingdom

George Athanassopoulos

Greece

Johann Auer

Austria

Pablo Avanzas

Spain

Alberto Avolio

Australia

Zsolt Bagi

United Kingdom

William Baker

United States of America

Ester Ballana

Spain

Lia Evi Bang

Denmark

Sripal Bangalore

United States of America

Iddo Ben-Dov

United States of America

William Benish

United States of America

Elizabeth Benito-Garcia

Portugal

Jacob Bentzon

Denmark

Marius Berman

United Kingdom

Fausto Biancari

Finland

Pawel Bieganowski

Poland

Georg Biesenbach

Austria

Grzegorz Bilo

Italy

Leif-Hendrik Boldt

Germany

Giuseppe Boriani

Italy

Emanuela Bostjancic

Slovenia

Clinton Brawner

United States of America

Jana Brguljan Hitij

Slovenia

Jean-Christophe Brisset

United States of America

Julie Brothers

United States of America

Carlos Brotons

Spain

Giuseppe Bruschi

Italy

Gavin Bryce

United Kingdom

Michael Buerke

Germany

Marc Buijsrogge

Netherlands

Lorraine Buis

United States of America

Michael Bursztyn

Israel

Joe Caputo

Singapore

Andres Carrillo

Greece

Joseph Casey

Canada

Michele Ceccarelli

Italy

Parinya Chamnan

Thailand

Kuei-Chuan Chan

Taiwan

Kee-Lung Chang

Taiwan

Chung-I Chang

Taiwan

Chu-Chih Chen

Taiwan

Zhong Chen

China

Szu-Chia Chen

Taiwan

Chen-Huan Chen

Taiwan

Chih-Cheng Chen

Taiwan

Patrick F Chinnery

United Kingdom

Mirjam Christ-Crain

Switzerland

Marco Ciccone

Italy

Halina Cichoz-Lach

Poland

Neree Claes

Belgium

Alexander Clark

Canada

Daniela Concolino

Italy

David Conen

Switzerland

Robert Considine

United States of America

Jean-Michel Constantin

France

Richard Cook

Canada

Xenophon Costeas

Greece

Thais Coutinho

United States of America

Alexi Crosby

United Kingdom

Veronica D ’ Annunzio

Argentina

Matthew Damasiewicz

Australia

Francisco Darrieux

Brazil

Kusal Das

India

Fabrizio D’ascenzo

Italy

Giovanni Davi

Italy

Mark de Belder

United Kingdom

Jaap Deckers

Netherlands

Christian Delles

United Kingdom

Patrick Dessein

South Africa

Gerry Devlin

New Zealand

Luca Di Vito

Italy

Valdo Jose Dias da Silva

Brazil

Javier Diez

Spain

Theodoros Dimitroulas

United Kingdom

Ana Djordjevic-Dikic

Serbia

Martin Donato

Argentina

Chiara Donfrancesco

Italy

Wenfei Dong

China

Adam Dorfman

United States of America

Athanasios Dritsas

Greece

Frank Dudbridge

United Kingdom

Tomasz Dziedzic

Poland

Charles Eaton

United States of America

Nils Edvardsson

Sweden

Mohammad El Garhy

Egypt

Seif El-Jack

New Zealand

Klaus Empen

Germany

Brigitte Escoubet

France

Paulo Roberto Evora

Brazil

Britt Falskov

Denmark

David Fedida

Canada

Trevor Ferguson

Jamaica

Elisabet Ferrer Andres

United Kingdom

Gerasimos Filippatos

Greece

Aloke Finn

United States of America

Bernard Fleury

France

Marco Francone

Italy

Juan Gagliardi

Argentina

Armen Yuri Gasparyan

United Kingdom

Carlo Gaudio

Italy

Carmine Gazzaruso

Italy

Omer Gedikli

Turkey

Welton Gersony

United States of America

Stefano Ghio

Italy

Juan Gimeno

Spain

Betti Giusti

Italy

Annie Gjelsvik

United States of America

Luca Gondoni

Italy

Anjelica Gonzalez

United States of America

German Gonzalez

Argentina

Matthias Götberg

Sweden

Shawn Gregory

United States of America

Liliana Grinfeld

Argentina

Sibel Guldiken

Turkey

Wayne Gulliver

Canada

Miguel Gus

Brazil

Atte Haarala

Finland

Christiane L Haase

Denmark

Martial Hamon

France

Yaling Han

China

John-Bjarne Hansen

Norway

Aly Hassan

Germany

Sean Hennessy

United States of America

Kwok-ming Ho

Australia

Willibald Hochholzer

Germany

Caisa Hofgren

Sweden

Tsuyoshi Honda

Japan

Benjamin Horne

United States of America

Mahmoud Houmsse

United States of America

Jie Huang

United Kingdom

Shu-chien Huang

Taiwan

Heikki Huikuri

Finland

Kuan-Yu Hung

Taiwan

Yuji Ikari

Japan

Allan Iversen

Denmark

Kazuhiro Izawa

Japan

Masaki Izumo

Japan

Vladimir Jakovljevic

Serbia

Veronica Jamnik

Canada

Jiann-Shing Jeng

Taiwan

Myung Ho Jeong

Korea, South

Wang Jianchub

China

Qing-Hua Jin

China

Yan Jinchuan

China

Albert Marni Joensen

Denmark

Tom Johnson

United Kingdom

Kate Jolly

United Kingdom

Erik Jørgensen

Denmark

Rohina Joshi

Australia

Jyh-Ming Jimmy Juang

Taiwan

Auni Juutilainen

Finland

Mayuko Kadono

Japan

Andreas Kalogeropoulos

United States of America

Kamilu Musa Karaye

Nigeria

Kaveh Karimzadehnajar

United States of America

Kazuomi Kario

Japan

Juan-Carlos Kaski

United Kingdom

Gabi Kastenmüller

Germany

Jens Kastrup

Denmark

Ryuichi Kawamoto

Japan

David Keeling

United Kingdom

Roya Kelishadi

Iran

Bryan Kestenbaum

United States of America

Patrick Kesteven

United Kingdom

Mohammad Khademloo

Iran

Ijaz Khan

United States of America

Helmut Klein

United States of America

Klaus Fuglsang Kofoed

Denmark

Grzegorz Kopec

Poland

Vasilios Kotsis

Greece

Nicholas George Kounis

Greece

Leonard Kritharides

Australia

Martin Krssak

Austria

Ruan Kruger

South Africa

Iolanthe Kruger

South Africa

Thomas Kümler

Denmark

Varant Kupelian

United States of America

Tatiana Kuznetsova

Belgium

Wen Ter Lai

Taiwan

Pier Lambiase

United Kingdom

Mark Lamotte

Belgium

David Lanfear

United States of America

Gaetano Lanza

Italy

Aleksandar Lazarevic

Bosnia and Herzegovina

Frans Leenen

Canada

Joris Lemson

Netherlands

Yan Li

China

Yongjun Li

China

Qin Li

China

Charalampos Liakos

Greece

Irene Lie

Norway

Cheng-Chieh Lin

Taiwan

Kuan Pin Lin

Taiwan

Yanan Liu

United States of America

Yanping Liu

Belgium

Zhenguo Liu

United States of America

Jennifer Logue

United Kingdom

Jakob Lonborg

Denmark

Eric Loucks

United States of America

Lucinda Low

United Kingdom

John Jenn-Yenn Lu

Taiwan

Carla Lubrano

Italy

Alessandro Lupi

Italy

Gregory Makris

United Kingdom

Simon Maltais

United States of America

Antonis Manolis

Greece

Jonathan Mant

United Kingdom

Themistoklis Maounis

Greece

Louise Maple-Brown

Australia

Carmelita Marcantoni

Italy

Keiran Mather

United States of America

Patrick Mathieu

Canada

Nilesh Mathuria

United States of America

Bunzo Matsuura

Japan

Jussi Mattila

Finland

Gearoid McMahon

United States of America

Coleen McNamara

United States of America

Rohit Mehta

United States of America

Nehal Mehta

United States of America

Pascal Meier

United States of America

Patrick Meimoun

France

Ofer Merin

Israel

Louis Messina

United States of America

Antonio Miceli

Italy

Carlos Miranda

Brazil

Saira Mirza

Netherlands

Deddo Moertl

Austria

Noushin Mohammadifard

Iran

Fabrizio Montecucco

Switzerland

Augusto Montezano

United Kingdom

Antonio Moretti

Italy

Mario Luca Morieri

Italy

Thomas Mueller

Austria

Siddharth Mukerji

United States of America

Giuseppe Mule

Italy

Elizabeth Muxfeldt

Brazil

Datshana Naidoo

South Africa

Franjo Naji

Slovenia

James Navalta

United States of America

Abboud Nesrine

France

Mario Neves

Brazil

Mark Noble

United Kingdom

Margareta Norberg

Sweden

Ntobeko Ntusi

United Kingdom

Evangelia Ntzani

Greece

Tomohiro Numata

Japan

José Pedro L. Nunes

Portugal

Altacilio Nunes

Brazil

Augustine Odili

Nigeria

Marc-Alexander Ohlow

Germany

JM Olivot

United States of America

Agnieszka Olszanecka

Poland

Steven Onkelinx

Belgium

Maksymilian Opolski

Poland

Petr Ostadal

Czech Republic

Petar Otasevic

Serbia

Luigi Palmieri

Italy

Pasquale Palmiero

Italy

Orestis Panagiotou

Greece

Vasileios Panoulas

United Kingdom

Ira Parness

United States of America

Giuseppe Passarino

Italy

Jeetesh Patel

United Kingdom

Sune Folke Pedersen

Denmark

Sanne A. E. Peters

Netherlands

Alessandro Petrolini

Italy

Rob Phillips

Australia

Daniel Pineiro

Argentina

Bertram Pitt

United States of America

Tudor Poerner

Germany

Don Poldermans

Netherlands

Luigi Politi

Italy

Toni Pollin

United States of America

Katrina Poppe

New Zealand

Tatjana Potpara

Serbia

Aleksander Prejbisz

Poland

Besim Prnjavorac

Bosnia and Herzegovina

Giuseppe Pugliese

Italy

Mariusz Pytkowski

Poland

Salah Qanadli

Switzerland

Tanja Raedle-Hurst

Germany

Shaoqi Rao

China

Rainer Rauramaa

Finland

Julie Redfern

Australia

Tobias Reichlin

Switzerland

Manja Reimann

Germany

Joahnnes Reitsma

Netherlands

Eun-Jung Rhee

Korea, South

Fruhling Rijsdijk

United Kingdom

Gilles Rioufol

France

Guillermo Rodrigo

Spain

Luis Rohde

Brazil

Minna Romano

Brazil

Ulrich Ronellenfitsch

Germany

Markus Roos

Germany

Adam Rose

United States of America

Laurent Roten

Switzerland

Alistair Royse

Australia

Zvonko Rumboldt

Croatia

Hans-Juergen Rupprecht

Germany

Alisdair Ryding

United Kingdom

Ligita Ryliskyte

Lithuania

Isao Saito

Japan

Uchechukwu Sampson

United States of America

David Samuels

United States of America

Valeria Sandrim

Brazil

Luca Santini

Italy

Nizal Sarrafzadegan

Iran

Motoji Sawabe

Japan

Peter Scarborough

United Kingdom

Vernon Schabert

United States of America

Bruno Scheller

Germany

Alexander Schirdewan

Germany

Axel Schlitt

Germany

Andre Schmidt

Brazil

Mikael Schneider

Denmark

Paul Scott

United Kingdom

Angelo Scuteri

Italy

Jitka Seidlerová

Czech Republic

Erik Serné

Netherlands

Arti Shah

United States of America

Chengxing Shen

China

Gong-Qing Shen

United States of America

Jae-Gook Shin

Korea, South

So-Youn Shin

Korea, South

Sandy Shiralkar

United Kingdom

Wang Shutang

China

Kou-Gi Shyu

Taiwan

Jefffrey Silbiger

United States of America

Gregg Silverman

United States of America

George Siontis

Greece

Peter Smars

United States of America

Osama Soliman

Netherlands

Jithendra Somaratne

Australia

Neal Sondheimer

United States of America

Kimon Stamatelopoulos

Greece

Paul Steendijk

Netherlands

Louis-Mathieu Stevens

Canada

Alexander Stokes

United States of America

Celine Straczek

France

Hasan Sunar

Turkey

Jay Suntharalingam

United Kingdom

Christian Terkelsen

Denmark

David Tester

United States of America

Ajit Thachil

India

Colleen Thomas

Australia

Margaret Thorogood

United Kingdom

Jens Jakob Thune

Denmark

Jani Tikkanen

Finland

Carlos Tirapelli

Brazil

Kirsten Tolstrup

United States of America

Christian Torp-Pedersen

Denmark

Mark Toshner

United Kingdom

Dimitris Tousoulis

Greece

Marek Treiman

Denmark

Jack Tsan

United States of America

Konstantinos Tsilidis

Greece

Pansy Tung

United States of America

Mohit Turagam

United States of America

Ioanna Tzoulaki

Greece

Martin Unverdorben

United States of America

Marcus Vallaster

United States of America

Cinzia Valzania

Italy

Lize van der Merwe

South Africa

Allard C van der wal

Netherlands

Mandy van Hoek

Netherlands

Tjeerd van Staa

United Kingdom

Olivier M van der Veken

Belgium

Marlien Vanrfield

Australia

Massimiliano Veroux

Italy

Wan Nazaimoon Wan Mohamud

Malaysia

Yongfei Wang

United States of America

Mohammad Wasay

Pakistan

Eiichi Watanabe

Japan

Peter Weeke

Denmark

Yingjie Wei

China

Gillian Whalley

New Zealand

James Wingrove

United States of America

Barbara Wizner

Poland

Wiktoria Wojciechowska

Poland

Christian Wollmann

Austria

Rafal Wolny

Poland

Sean Wu

United States of America

Qingyu Wu

China

Andrzej Wykretowicz

Poland

Panagiotis Xaplanteris

Greece

Quansheng Xingqs

China

Takumi Yamada

United States of America

Eric Yang

United States of America

Joseph Yeboah

United States of America

Hung-I Yeh

Taiwan

Sung Sug (Sarah) Yoon

United States of America

Tomasz Zapolski

Poland

Claudia Zemmrich

Germany

Zhenxiang Zhao

United States of America

Xingwei Zhe

China

Paul Ziegler

United States of America

Leonardo Zornoff

Brazil

## Pre-publication history

The pre-publication history for this paper can be accessed here:

http://www.biomedcentral.com/1471-2261/13/14/prepub

